# Visceral artery aneurysms: evolving interdisciplinary management and future role of the abdominal surgeon

**DOI:** 10.1186/s40001-019-0374-9

**Published:** 2019-02-28

**Authors:** Vittorio Branchi, Carsten Meyer, Frauke Verrel, Alexander Kania, Edwin Bölke, Alexander Semaan, Arne Koscielny, Jörg C. Kalff, Hanno Matthaei

**Affiliations:** 1 Department of General, Visceral, Thoracic and Vascular Surgery, University Hospital Bonn, Sigmund-Freud-Strasse 25, 53127 Bonn, Germany; 2 Department of Radiology, University Hospital Bonn, Sigmund-Freud-Strasse 25, 53127 Bonn, Germany; 3 Department of Radiotherapy and Radiation Oncology, Faculty of Medicine, Henirich-Heine Universität, Moorenstrasse 5, 40225 Düsseldorf, Germany

**Keywords:** Visceral artery aneurysms, Hemorrhage, Emergency bleeding, Interventional radiology, Open surgery

## Abstract

**Background:**

Visceral artery aneurysms (VAA) are rare vascular lesions. Clinically silent VAA are increasingly detected by cross-sectional imaging but some lesions are at risk for rupture with severe bleeding. The aim of the present study was to evaluate the trends in the interdisciplinary management at a tertiary center.

**Methods:**

Patients who underwent treatment for VAA at University Hospital of Bonn between 2005 and 2018 were enrolled in this retrospective study. Demographic, clinical, VAA-specific data as well as information on therapy, early and long-term outcome were collected and statistically analyzed.

**Results:**

Forty-two consecutive patients, 19 females and 23 males with a median age of 59 years (range 30–91 years), were diagnosed with 56 VAA. The majority were true aneurysms (*N* = 32; 57%), whereas 43% (*N* = 24) were pseudoaneurysms. The most common localization was the splenic artery (*N* = 18; 32%) and the average diameter was 3 cm (range 1–5 cm). Twenty-five patients (59.5%) had VAA-related symptoms such as chronic abdominal pain and hemorrhage at primary diagnosis, while the diagnosis was incidental in 17 patients (40.5%). Eleven patients (26%) underwent open surgery whereas 29 patients (69%) received an endovascular treatment. Patients with pseudoaneurysms were significantly older (*P *= 0.003), suffered more often from associated symptoms (*P *< 0.001) and required more emergency interventions (*P *< 0.0001) compared to those with true VAA. In the last years, the number and proportion of true VAA increased significantly (*P *< 0.001) while a significantly larger proportion could be managed interventionally (*P *= 0.017).

**Conclusions:**

VAA are increasingly detected on imaging with lesions presenting very heterogeneously. Due to the risk of lethal rupture and in the absence of reliable prognostic markers, all the patients with VAA should be offered definite treatment. Localization, anatomy and the end-organ perfusion after intervention or operation are the most important aspects to consider when planning a treatment for VAA. For this reason, a multidisciplinary evaluation of every individual patient is necessary for an optimized outcome.

## Introduction

Visceral artery aneurysms (VAA) are rare yet serious vascular lesions. Their incidence has been rising over the last decades, largely due to the demographic shift and the wide-spread use of cross-sectional imaging [1, 2]. VAA may arise in every splanchnic artery and are either classified into true aneurysms (TVAA) or pseudoaneurysms (PVAA) [3]. True aneurysms consist of abnormal arterial wall dilatation of more than 1.5 times its normal diameter involving all three vascular layers [3]. Pseudoaneurysms are characterized by disruption of the media and intimal layers of the arterial wall, resulting in a confined hematoma with connection to the arterial lumen [4]. VAA etiopathogenesis is still not completely understood. Risk factors include collagen-related diseases such as Ehlers–Danlos syndrome or non-atherosclerotic, non-inflammatory diseases of the blood vessels like fibromuscular dysplasia [5, 6]. Interestingly, atherosclerosis seems to play only a marginal pathogenic role in aneurysm genesis [7]. The most common VAA site is the splenic artery, with a relative incidence of about 60%, followed by hepatic artery aneurysms representing approximately 20% [8–10]. Nowadays, VAA are increasingly diagnosed as incidental findings on CT, MRI or angiography imaging studies [11, 12]. Hemodynamic instability may occur due to intraabdominal bleeding in the event of VAA perforation, which is associated with a high mortality of 20–100% [12, 13].

Most symptomatic VAA patients presenting to the emergency room complain about abdominal pain and are, therefore, initially triaged for surgical evaluation. In case of severe intraabdominal hemorrhage due to perforation, an immediate emergency laparotomy might be inevitable to control the bleeding. Therefore, basic skills in vascular surgery are necessary for every surgeon performing emergency explorations in a hemodynamic instable patient. In fact, conventional surgery has been the standard of care in symptomatic as well as asymptomatic VAA for decades. Development and constant improvement of minimal invasive endovascular treatment have enriched the therapeutic repertoire for VAAs but also changed the therapeutic approach drastically. However, VAA are heterogeneous and a therapeutic approach has to be individualized for every patient by a multidisciplinary team including abdominal and vascular surgeons as well as interventional radiologists [3, 11]. The aim of this study was to evaluate the evolving interdisciplinary treatment of VAA at our tertiary center.

## Methods

### Patients

Patients with VAA diagnosis (ICD I72.8) admitted between 08/2005 and 08/2018 at the University Hospital of Bonn were included in this unicentric retrospective study. Ethical standards of the University of Bonn were fully acknowledged. Every patient signed informed consent before any kind of procedure. Patients with renal artery aneurysms were excluded. Elective treatment decisions were discussed for hemodynamically stable patients in a weekly interdisciplinary vascular board. In the event of VAA rupture with acute bleeding, interdisciplinary consultation was performed in the emergency room to decide for the most appropriate immediate treatment. The interdisciplinary team in both elective and emergency settings always included an abdominal surgeon, a vascular surgeon as well as an interventional radiologist. Pseudonymized data on patients, surgical and interventional therapy as well as on VAA characteristics were collected and analyzed. For the latter, an experienced diagnostic and interventional radiologist (CM) reviewed all relevant imaging studies and precisely measured and reclassified each VAA. To identify a possible change of treatment over the entire study period from 2005–2018, two subgroups were defined comprising patients presenting before (“early” group) and after January 1st, 2013 (“recent” group). The cutoff was chosen arbitrarily. Data concerning early outcomes (i.e., minor and major perioperative/interventional complications, hospital stay, and 30-day mortality) as well as long-term performance (i.e., overall survival and disease-related chronic morbidities) were reviewed. Complications were ranked according to the Clavien–Dindo classification [14].

### Statistics

Descriptive statistics (i.e., median, range, mean and standard deviation). Chi square test or *T* test was used where appropriate. Analyses were performed with the software SPSS Statistics (IBM, Armonk, New York, USA). A *P *< 0.05 was considered as statistically significant.

## Results

### Patients and outcome

**Fig. 1:**
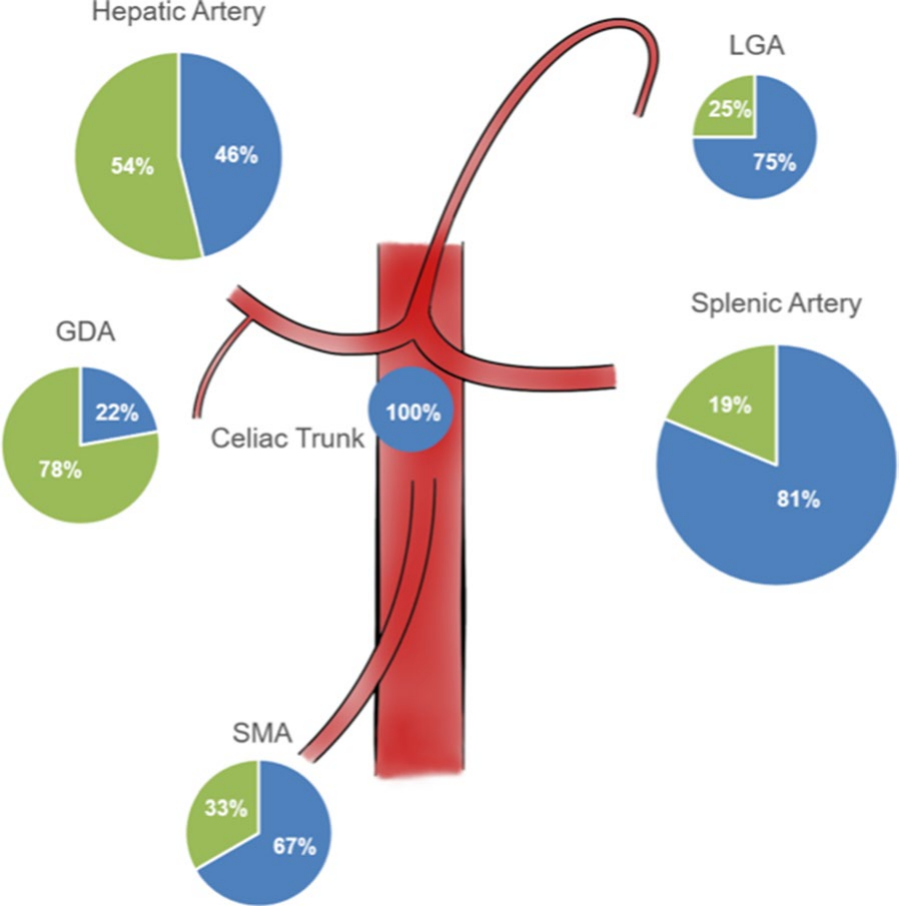
Relative frequencies of true VAA (blue) and pseudoaneuryms (green) according to their localization. *SMA* superior mesenteric artery, *GDA* gastroduodenal artery, *LGA* left gastric artery

During the study period, a total of 56 VAA in 42 consecutive patients including 19 females and 23 males with a median age of 59 years (range 30–91 years) were diagnosed with VAA. Detailed data are shown in Table 1. Of those, 17 Patients (40%) had an acute abdominal hemorrhage at the time of initial diagnosis, and 8 patients (19%) had a history of chronic abdominal pain. While 34 patients had a solitary VAA, 8 patients had multiple synchronous VAA in up to 5 different localizations. Median VAA size was 3 cm (range 1–5 cm). The most common VAA location was the splenic artery (*N* = 18; 32%), followed by hepatic artery aneurysms (*N* = 13; 23%) (Fig. 1). A total of 11 patients (26%) underwent an open surgical operation, whereas 29 patients (69%) were treated with an endovascular procedure.

**Table 1:** Patients and VAA characteristics according to the clinical presentation

N of patients	Total	SVAA	IVAA	
	42	25	17	
Age median (range)	59 (30–91)	63 (30–91)	58 (41–74)	*P *= 0.32
Female* n* (%)	19 (45%)	9 (36%)	10 (59%)	*P *= 0.15
Male * n* (%)	23 (55%)	16 (64%)	7 (41%)	
Diameter in cm mean (SD)	3 (± 2.1)	2.5 (± 1.5)	3.6 (± 2.5)	*P *= 0.37
Symptoms * n* (%)	25 (59.5%)	25 (100%)	0 (0%)	
Abdominal pain * n* (%)	8 (19%)	8 (32%)	0 (0%)	
Intraabdominal hemorrhage * n* (%)	17 (40.5%)	17 (68%)	0 (0%)	

N of aneurysms	56	34	22	
Splenic artery * n* (%)	18 (32%)	7 (21%)	11 (50%)	*P *= 0.14
Hepatic artery * n* (%)	13 (23%)	10 (29%)	3 (14%)	
Superior mesenteric artery * n* (%)	9 (16%)	5 (15%)	4 (18%)	
Gastroduodenalis artery * n* (%)	9 (16%)	7 (21%)	2 (9%)	
Left gastric artery * n* (%)	4 (7%)	2 (6%)	2 (9%)	
Celiac trunk * n* (%)	3 (5%)	3 (9%)	0 (0%)	
N of true aneurysms	32 (57%)	13 (38%)	19 (86%)	*P = 0.0002*
N of pseudoaneurysms	21 (37.5%)	20 (59%)	1 (4%)	
Indetermined morphology	3 (5%)	1 (3%)	2 (9%)	
Operation * n* (%)	11 (26%)	5 (20%)	6 (35%)	*P *= 0.25
Intervention * n* (%)	29 (69%)	19 (76%)	10 (59%)	
Coiling * n* (%)	23 (54.5%)	14 (56%)	9 (53%)	*P *= 0.28
Stenting * n* (%)	4 (9.5%)	4 (16%)	0 (0%)	
Stenting + coiling * n*(%)	2 (5%)	1 (4%)	1 (6%)	
No operation/intervention* n* (%)	2 (5%)	1 (4%)	1 (6%)	*P *= 0.76
Complications* n* (%)	17 (40.5%)	11 (44%)	6 (35%)	*P *= 0.68
Dindo 1	2 (5%)	0 (0%)	2 (12%)	*P *= 0.15
Dindo 2	8 (19%)	5 (20%)	3 (18%)	
Dindo 3	5 (12%)	4 (16%)	1 (6%)	
Dindo 4	2 (5%)	2 (8%)	0 (0%)	
Median hospital stay in days (range)	15 (1–238)	18 (1–238)	15 (2–43)	*P *= 0.14
N of deceased patients	9 (21%)	8 (32%)	1 (6%)	*P = 0.043*
Median follow-up in months (range)	10.5 (0–151)	11 (0–151)	10 (1–130)	*P *= 0.28

*IVAA* incidental visceral artery aneurysm, *SVAA* symptomatic visceral artery aneurism, *SD* standard deviation

Italic values indicate statistical significance (*P*<0.05)

**Fig. 2:**
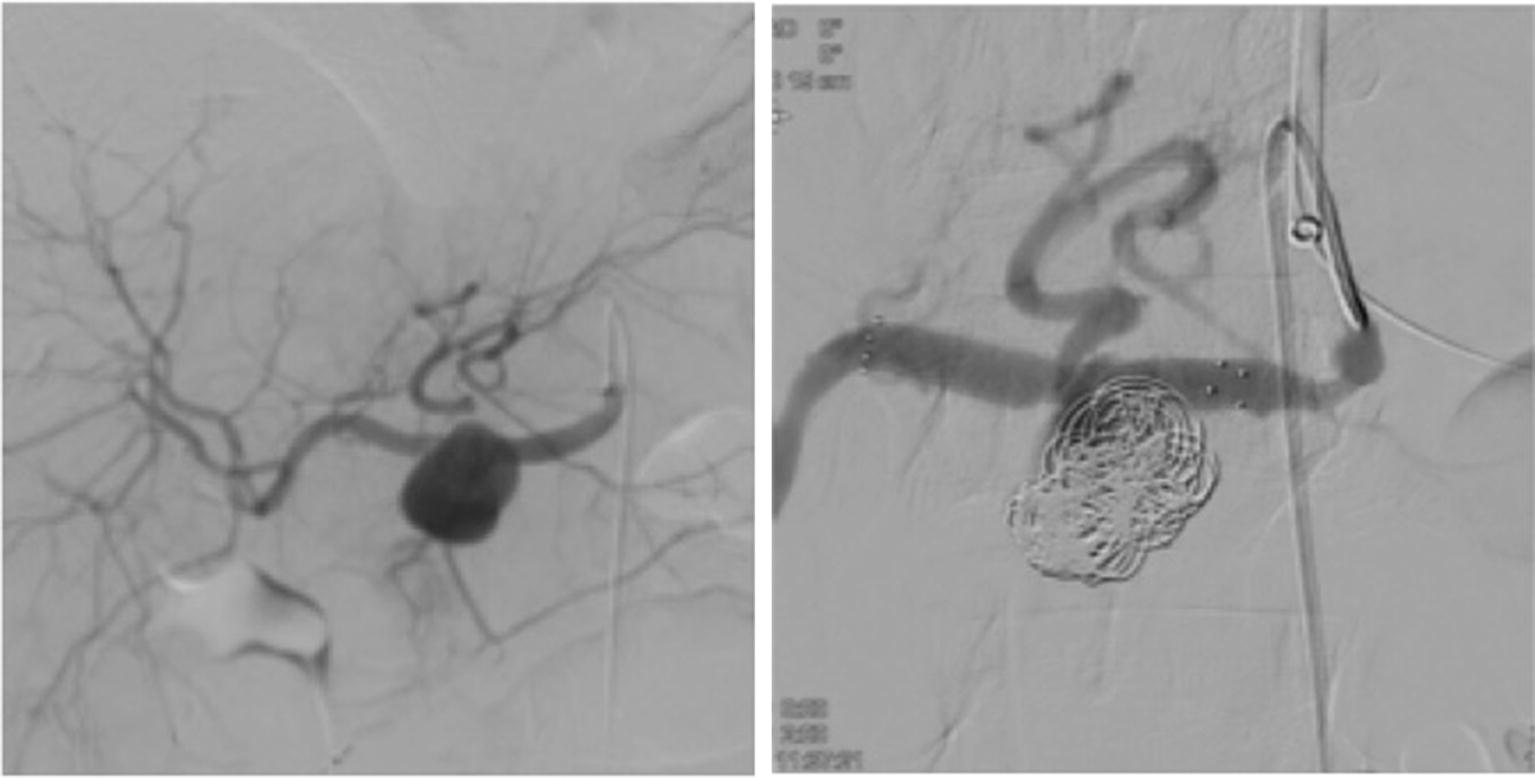
A 3.5-cm aneurysm of the hepatic artery before (left) and after (right) a combined radiological intervention with stenting and coiling

**Fig. 3:**
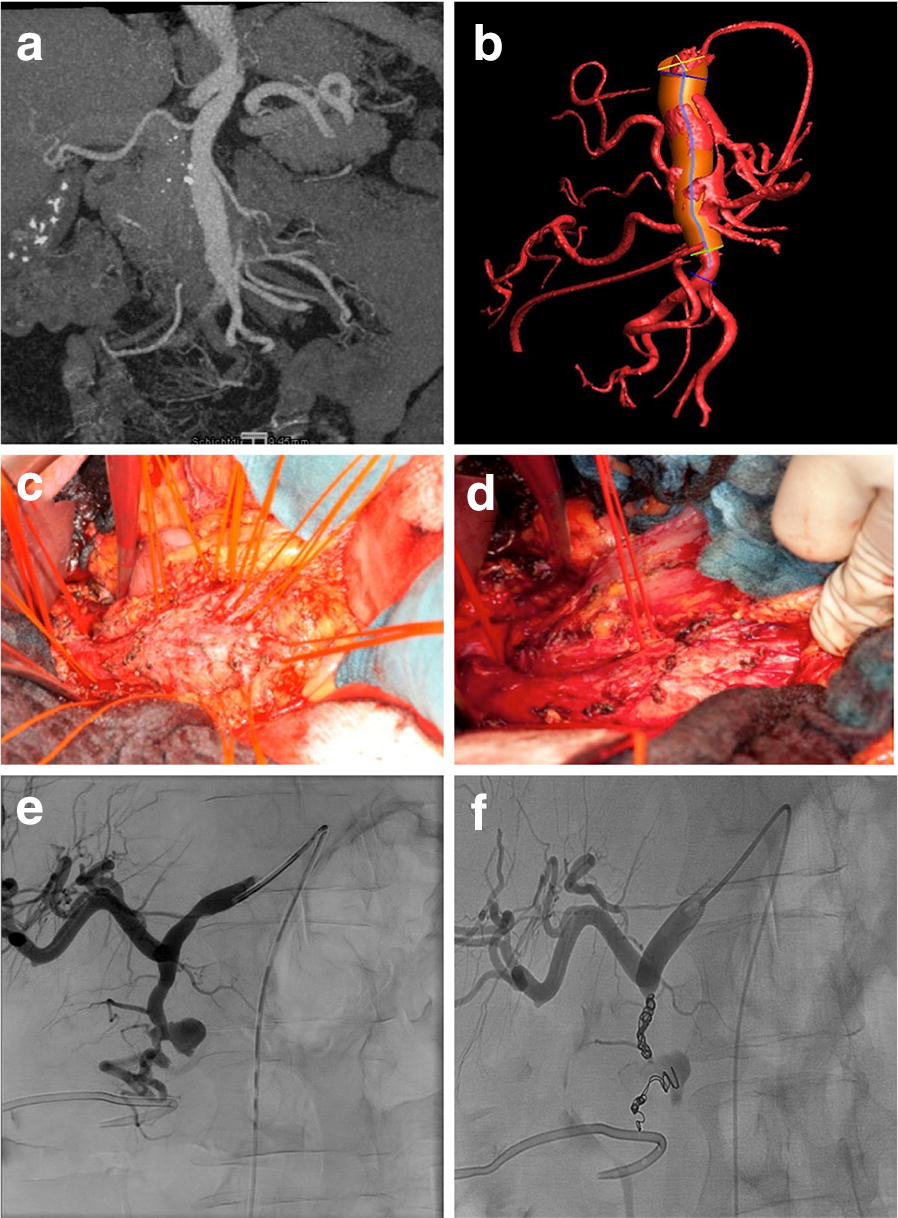
CT scan of a 62-year-old patient with a big asymptomatic SMA aneurysm (**a**). Digital reconstruction of the CT scan in **a** (**b**). Intraoperative images of the same patient before (**c**) and (**d**) after isolation of 14 collateral branches. Angiography of a 37-year-old patient with an aneurysm of the gastroduodenal artery after perforation (**e**). Same patient in **e** after a successful coiling procedure (**f**)

In particular, aneurysmorrhaphy was performed in four patients (9.5%); aneurysm resection with autologous vein patch reconstruction was performed in three patients (7%); two patients underwent splenectomy (5%) and aneurysm resection with end-to-end anastomosis and aneurysm resection with synthetic graft interposition was performed in one patient (2%), respectively. Among the endovascular procedures, coiling was performed in 23 patients (54.5%), stenting in 4 patients (9.5%), and a combination of stenting and coiling in 2 patients (5%) (Figs. 2 and 3).

Complication rate was 40.5% (*N* = 17) mostly including Clavien–Dindo Grade 2 adverse events. The median hospital stay was 15 days (range 1–238 days). Median follow-up was 10.5 months (0–151 months) and nine patients died (21%). Only one patient died in strict association of his VAA (2%).

### Conservative management of two VAA patients

Until 2012, every patient diagnosed with VAA has been treated either surgically or interventionally. Since 2013, two patients (5%) with treatment indication did not receive any VAA-specific therapy. The first patient had a 3.1-cm aneurysm of the hepatic artery and a 3.6-cm aneurysm of the left gastric artery, both incidentally detected. The patient refused any kind of intervention and only presented to the first follow-up at 2 months after primary diagnosis, when the aneurysms size and morphology proved stable. Thereafter, the patient refused any further follow-up evaluations. The second patient had a 2-cm aneurysm of the celiac trunk and a 1.3-cm aneurysm of the superior mesenteric artery both of which were asymptomatic. A radiological intervention was contraindicated due to the site and anatomy of the aneurysms, and the patient refused surgery. During 37 months of regular follow-up (every 2 months for the first year, every 4 months during the second year and twice a year thereafter), both VAA remained stable and asymptomatic.

### Asymptomatic versus symptomatic VAA

A total of 17 patients (40%) had no history of VAA-related symptoms and were incidentally detected (IVAA) while 25 patients (60%) had VAA-related symptoms at the time of primary diagnosis (SVAA). PVAA were more frequently found in the SVAA group (*N* = 20; 59% vs. *N* = 1; 4%; *P *= 0.0002) and these patients had a higher mortality (*N* = 8; 32% vs. *N* = 1; 6%; *P *= 0.043). Both groups were similar, however, regarding the parameters age, gender, maximum diameter of VAA, type of intervention, complication rate and duration of hospital stay.

### True aneurysms versus pseudoaneurysms

Among the 56 VAA, a majority of 57% were true aneurysms (TVAA: *N* = 32), whereas 37% were pseudoaneurysms (PVAA: *N* = 17). Three VAA could not be assigned to either group based on radiological or pathological assessments. Characteristics are summarized in Table 2. Patients with PVAA were significantly older compared to patients with TVAA (median 72 years vs. 57 years; *P *= 0.003) and suffered more often from symptoms such as abdominal pain (*N* = 16; 94% vs. *N* = 8; 36%; *P *= 0.0002). Localization of VAA differed in both groups since TVAA were typically localized in the splenic artery (*N* = 13; 41%), whereas PVAA were mainly localized in the hepatic (*N* = 7; 33%) and gastroduodenal artery (*N* = 7; 33%). Emergency treatment was more frequently necessary in the PVAA group (*N* = 15; 88% vs. *N* = 1; 4.5%; *P *< 0.0001). In addition, hospital stay of these patients was significantly longer (22 days vs. 15 days; *P *= 0.028) and their mortality rate higher (*N* = 7; 41% vs. *N* = 2; 9%; *P *= 0.018). Both groups were, however, similar regarding the parameters gender, maximum diameter of VAA, and type of intervention.

**Table 2:** Patients and VAA characteristics according to the aneurysm morphology

N of patients	True VAA	Pseudo VAA	
	22	17	
Age median (range)	57 (30–79)	72 (36–91)	*P = 0.003*
Female * n* (%)	12 (45.5%)	11 (65%)	*P *= 0.52
Male * n* (%)	10 (55.5%)	6 (35%)	
Diameter in cm mean (SD)	3 (± 1.6)	2 (± 1.3)	*P *= 0.10
Symptoms * n* (%)	8 (36%)	16 (94%)	*P = 0.0002*
Abdominal pain * n* (%)	7 (31%)	1 (6%)	*P < 0.0001*
Intraabdominal hemorrhage * n* (%)	1 (5%)	15 (88%)	

N of aneurysms	32	21	
Splenic artery * n* (%)	13 (41%)	3 (14%)	*P = 0.046*
Hepatic artery * n* (%)	6 (19%)	7 (33%)	
Superior mesenteric artery * n* (%)	6 (19%)	3 (14%)	
Left gastric artery * n* (%)	3 (9%)	1 (5%)	
Gastroduodenalis artery * n* (%)	2 (6%)	7 (33%)	
Celiac trunk * n* (%)	2 (6%)	0 (0%)	
Emergency * n* (%)	1 (4.5%)	15 (88%)	*P < 0.0001*
Elective * n* (%)	21 (95%)	2 (12%)	
Operation * n* (%)	5 (23%)	3 (18%)	*P *= 0.58
Intervention * n* (%)	15 (68%)	14 (82%)	
Coiling * n* (%)	13 (59%)	10 (59%)	*P *= 0.51
Stenting * n* (%)	1 (4.5%)	3 (18%)	
Stenting + coiling *n* (%)	1 (4.5%)	1 (6%)	
No operation/intervention* n* (%)	2 (9%)	0 (0%)	*P *= 0.20
Complications* n* (%)	8 (36%)	7 (41%)	*P *= 0.073
Dindo 1* n* (%)	2 (9% %)	0 (0%)	*P *= 0.25
Dindo 2* n* (%)	3 (14%)	3 (18%)	
Dindo 3* n* (%)	3 (14%)	2 (12%)	
Dindo 4* n* (%)	0 (0%)	2 (12%)	
Median hospital stay in days (range)	15 (2–43)	22 (1–238)	*P = 0.0028*
Median follow-up in months (range)	13 (1–112)	2 (0–59)	*P *= 0.054
N of deceased patients	2 (9%)	7 (41%)	*P = 0.018*

Three VAA could not be assigned to either group based on radiological or pathological assessments. *VAA* visceral artery aneurysm, *SD* standard deviation

Italic values indicate statistical significance (*P*<0.05)

### VAA treatment and outcome in the early versus recent period

Among the 42 patients, 17 were diagnosed before (40%) and 25 after January 1st, 2013 (60%) (Table 3). Patients in the recent treatment group where significantly younger than those in the earlier group (median age 58 years vs. 71 years; *P *= 0.01). Furthermore, there was a significantly higher rate of true aneurysms detected during more recent years (*N* = 26; 76% vs. *N* = 6; 27%; *P *= 0.0006). In the early period, 47% of the patients underwent primary surgery for VAA (*N* = 8), whereas 53% were treated with an endovascular procedure (*N* = 9). More recently, however, a vast majority of patients were treated with radiological intervention (*N* = 20; 80% vs. *N* = 5; 20%), and this shift in therapeutic strategy proved to be statistically significant (*P *= 0.017). Patients’ characteristics in the early and in the recent treatment group were, however, similar in terms of gender, clinical presentation, VAA dimensions and localization, complication rate and hospital stay.

**Table 3:** Patients and VAA characteristics according to the period of treatment

N of patients	Before 2013	Since 2013	
	17	25	
Age median (range)	71 (47–91)	58 (30–80)	*P = 0.01*
Female* n* (%)	6 (35%)	13 (52%)	*P *= 0.29
Male* n* (%)	11 (65%)	12 (48%)	
Diameter in cm mean (SD)	2.9 (± 1.5)	2.6 (± 1.5)	*P *= 0.52
Symptoms* n* (%)	11 (65%)	14 (41%)	*P *= 0.57
Abdominal pain* n* (%)	3 (18%)	5 (15%)	*P *= 0.65
Intraabdominal hemorrhage* n* (%)	8 (47%)	9 (26%)	

N of aneurysms	22	34	
Splenic artery* n* (%)	5 (23%)	13 (38%)	*P *= 0.76
Hepatic artery* n* (%)	6 (27%)	7 (21%)	
Superior mesenteric artery* n* (%)	5 (23%)	4 (12%)	
Gastroduodenalis artery* n* (%)	3 (14%)	6 (18%)	
Left gastric artery* n* (%)	2 (9%)	2 (6%)	
Celiac trunk* n* (%)	1 (5%)	2 (6%)	
N of true aneurysms	6 (27%)	26 (76%)	*P = 0.0006*
N of pseudoaneurysms	13 (59%)	8 (24%)	
Indetermined morphology	3 (14%)	0 (0%)	
Operation* n* (%)	8 (47%)	3 (12%)	*P = 0.017*
Intervention * n* (%)	9 (53%)	20 (80%)	
Coiling * n* (%)	7 (41%)	16 (64%)	*P *= 0.45
Stenting * n* (%)	2 (12%)	2 (8%)	
Stenting + coiling* n* (%)	0 (0%)	2 (8%)	
No operation/intervention* n* (%)	0 (0%)	2 (8%)	*P *= 0.23
Emergency	8 (47%)	9 (36%)	*P *= 0.62
Elective	9 (53%)	14 (56%)	
Complications * n* (%)	9 (53%)	8 (32%)	*P *= 0.17
Dindo 1	0 (0%)	2 (8%)	*P *= 0.25
Dindo 2	6 (35%)	2 (8%)	
Dindo 3	2 (12%)	3 (12%)	
Dindo 4	1 (6%)	1 (4%)	
Median hospital stay in days (range)	16 (1–238)	13 (2–122)	*P *= 0.61
Median follow-up in months (range)	37 (0–151)	7 (0–46)	*P = 0.0007*
N of deceased patients	2 (12%)	7 (28%)	*P *= 0.21

*VAA* visceral artery aneurysm, *SD* standard deviation

Italic values indicate statistical significance (*P*<0.05)

## Discussion

The use of diagnostic cross-sectional imaging has drastically increased in the last decades, particularly in high-volume cancer and emergency centers [15–17]. This, in combination with the ongoing demographic shift in Western countries appears to be responsible for the increasing incidence of VAA as previously described [18, 19]. Although this vascular disorder is undoubtedly rare, we noticed a slight increase in VAA incidence over the last 13 years, which specifically encouraged us to conduct this retrospective study.

A VAA often presents as acute abdomen with life-threatening hemorrhage. Approximately, 40% of our patients suffered from intraabdominal bleeding, mostly in combination with hemorrhagic shock. This high rate of VAA perforation at primary diagnosis noticed in our cohort is comparable to previously described incidence rates [1, 2, 11, 20]. Our data furthermore confirmed that especially pseudoaneurysms carry an extremely high risk for rupture. This might be related to their wall instability compared to true VAA. We observed pseudoaneurysms occasionally after major abdominal surgery such as hepatobiliary and pancreatic tumor resections. This dangerous condition tends to occur in elderly patients in the first postoperative days. In particular, pseudoaneurysms can develop after pancreatic leakage or pancreatitis because of enzymatic digestion of the arterial wall while any “index bleeding” from abdominal drains should be considered for immediate contrast-enhanced CT scan.

Due to the potentially fatal consequences of VAA perforation, there has been an effort to identify reliable risk factors predicting aneurysm growth and rupture. Undoubtedly, posttraumatic pseudoaneurysms require instant treatment to avoid severe morbidity and lethal courses. In contrast to pseudoaneurysm, the natural history of true VAA and their possible progression over time is hard to predict. Hence, there is an ongoing debate about indication and the right timing of therapy especially in the absence of VAA-related symptoms. Due to small retrospective studies and the complete absence of prospective randomized trial, the evidence level regarding VAA treatment is low. Nevertheless, pregnancy is the only established risk factor for rupture in patients with true splenic aneurysms. Aneurysms of this location, more frequent in females (4:1), are even four times more common in multiparous women owing to hormone-related vascular changes [21, 22]. For this reason, it has been proposed that all incidentally detected aneurysms of the splenic artery during pregnancy, or even in women of childbearing age, should be promptly treated [23, 24]. Aside from VAA type and location, there has been an ongoing debate about size of true VAA with respect to timing of treatment. According to several expert opinions, all true aneurysm > 2 cm in maximum diameter must be treated [4, 25, 26], which is in accordance with the guidelines defined by the German Association for Vascular Surgery (Deutsche Gesellschaft für Gefäßchirurgie, DGG) [27]. However, due their unpredictable growth, therapy of every VAA irrespective of their diameter is encouraged according to DGG recommendations. Furthermore, an endovascular approach should be aimed for. Still, an emergency laparotomy needs to be performed in case of VAA rupture or if an interventional procedure does not appear to be a safe option or is unavailable. In selected patients, a watchful waiting approach may occasionally be justified. For example, in the case of severe comorbidities and morphologically stable, small TVAA, a conservative treatment may be considered.

There is a constant ambition to improve both surgical and interventional techniques to ensure the least invasive procedure for every VAA detected. As reported, in recent years a shift has been noticed towards a therapeutic paradigm prioritizing radiological intervention over surgery. The question of whether VAA should be managed by open surgery or endovascular treatment has never been addressed by randomized studies, and only small retrospective cohorts have been published. Cochennec et al. [28] compared open and endovascular repairs in two European institutions over a 15-year period in a retrospective study. Sixteen patients were treated by open repair, and 15 patients by endovascular procedures. The authors noticed no significant difference between open repair and endovascular therapy in terms of 30-day mortality rate and perioperative complications. Marone et al. [29] compared surgical and endovascular treatment in a cohort of 94 consecutive patients. Sachdev et al. [30] compared 24 patients with surgical repair to 35 who underwent endovascular treatment. The authors reported an 89% success rate following coil embolization or stent-graft therapy confirming the efficacy of endovascular treatment for VAA, which was comparable to results in the surgical cohort. Paralleling these findings, the data from our center are encouraging with respect to both the safety and the efficacy of radiological procedures thereby reaching success rates after surgery with a tendency to shorter hospital stays. Despite these findings, the surgeon will continue to play a central role in the management of complicated VAA reflected by 25% of all and 12% of recent VAA patients needing open surgery. Aside from hemorrhage control and basic vascular reconstruction, the abdominal surgeon should critically and constantly evaluate the end-organ perfusion. In fact, in case of inadequate vascular supply, the abdominal surgeon should promptly perform procedures such as bowel resections, splenectomy or partial liver resections.

### Implications, perspective and limitations of the study

The aim of the present study was to evaluate the trend in the interdisciplinary VAA management at a tertiary center. We retrospectively analyzed a consecutive series of 42 VAA patients over a period of more than 10 years. One of the main practical contributions of the present research is that it underlines the shift toward an endovascular therapeutic paradigm during the last few years with excellent short- and long-term outcomes. In addition, this study highlighted the substantial differences between PVAA and TVAA in terms of outcome. In fact, symptomatic VAA are mainly PVAA and incidental VAA are mainly TVAA. This should help an interdisciplinary team through the decision process to identify high-risk patients, therefore, orienting toward an early elective intervention.

Our study has some limitations. Inherent to its retrospective design, we cannot draw valid conclusions regarding optimal treatment of true VAA and pseudoaneurysms. Ideally, prospective multicenter randomized trials are required to clearly define the optimal treatment for VAA. However, it is questionable whether a conservative treatment arm can be ethically justified due to the high morbidity and mortality associated with VAA perforation and other VAA-related complications such as end-organ ischemia.

## Conclusion

VAA is a rare vascular disease. Nonetheless, it should be considered as a differential diagnosis in the event of acute abdomen, in particular in the presence of hemodynamic instability. Abdominal and vascular surgeons should be aware of the therapeutic options in case an endovascular procedure is contraindicated or unavailable. Abdominal surgeons should be prepared to handle both, emergencies involving VAA rupture and also complicated cases, in which an intervention does not represent the definitive therapy. A multidisciplinary evaluation is the key to an appropriate treatment allocation in this heterogeneous disease. An evaluation at a tertiary center with high expertise in visceral and vascular surgery and interventional radiology is mandatory. A multi-institutional registry and, at best, prospective trials might help improve diagnostic strategies and identify prognostic factors to eventually establish evidence-based guidelines for VAA management.

## Abbreviations

VAA: visceral artery aneurysms
TVAA: true aneurysms
PVAA: pseudoaneurysms
IVAA: incidental visceral artery aneurysms
SVAA: symptomatic visceral artery aneurysms
CT: computed tomography
MRI: magnetic resonance imaging
DGG: Deutsche Gesellschaft für Gefäßchirurgie—German Association for Vascular Surgery
